# The Novel Anticancer Aryl-Ureido Fatty Acid CTU Increases Reactive Oxygen Species Production That Impairs Mitochondrial Fusion Mechanisms and Promotes MDA-MB-231 Cell Death

**DOI:** 10.3390/ijms251910577

**Published:** 2024-10-01

**Authors:** Stanton Tam, Balasubrahmanyam Umashankar, Md Khalilur Rahman, Hassan Choucair, Tristan Rawling, Michael Murray

**Affiliations:** 1 Pharmacogenomics and Drug Development Group, Sydney Pharmacy School, Faculty of Medicine and Health, University of Sydney, Sydney, NSW 2006, Australia; stanton.tam@anu.edu.au (S.T.); b.umashankar@unsw.edu.au (B.U.); khalilur@mucpharm.com (M.K.R.); hassan.choucair@nd.edu.au (H.C.); 2 School of Mathematical and Physical Sciences, Faculty of Science, University of Technology Sydney, Ultimo, NSW 2007, Australia; tristan.rawling@uts.edu.au

**Keywords:** aryl-ureido fatty acids, anticancer agent, mitochondrial membrane, reactive oxygen species, mitochondrial fission and fusion

## Abstract

Cancer cell mitochondria are functionally different from those in normal cells and could be targeted to develop novel anticancer agents. The aryl-ureido fatty acid CTU (16({[4-chloro-3-(trifluoromethyl)phenyl]-carbamoyl}amino)hexadecanoic acid) is the prototype of a new class of targeted agents that enhance the production of reactive oxygen species (ROS) that disrupt the outer mitochondrial membrane (OMM) and kill cancer cells. However, the mechanism by which CTU disrupts the inner mitochondrial membrane (IMM) and activates apoptosis is not clear. Here, we show that CTU-mediated ROS selectively dysregulated the OMA1/OPA1 fusion regulatory system located in the IMM. The essential role of ROS was confirmed in experiments with the lipid peroxyl scavenger α-tocopherol, which prevented the dysregulation of OMA1/OPA1 and CTU-mediated MDA-MB-231 cell killing. The disruption of OMA1/OPA1 and IMM fusion by CTU-mediated ROS accounted for the release of cytochrome c from the mitochondria and the activation of apoptosis. Taken together, these findings demonstrate that CTU depolarises the mitochondrial membrane, activates ROS production, and disrupts both the IMM and OMM, which releases cytochrome c and activates apoptosis. Mitochondrial-targeting agents like CTU offer a novel approach to the development of new therapeutics with anticancer activity.

## 1. Introduction

Mitochondrial ATP synthesis and apoptosis are dysregulated in cancer cells, and new classes of anticancer agents could be developed that act by targeting that organelle [1]. Structurally, the mitochondrion consists of inner and outer mitochondrial membranes (IMM and OMM, respectively) that enclose the inter-membrane space [2]. The mitochondrial electron transport chain (ETC) utilises a series of multiprotein complexes that are located in the IMM and generate ATP by oxidative phosphorylation [3]. In the process, protons are shunted by ETC complexes I, III, and IV into the inter-membrane space, which establishes electrochemical and proton gradients across the IMM that are used to drive ATP synthesis [3]. A number of chemicals are able to uncouple oxidative phosphorylation by transferring protons from the inter-membrane space back across the IMM, which decreases their availability for ATP synthesis [4]. We recently reported that the novel aryl ureido-fatty acid derivative CTU targets cancer cell mitochondria and disrupts ATP production and cell viability by acting as a protonophore that uncouples electron transport [5].

The mitochondrion is also an important cellular source of reactive oxygen species (ROS) that are generated during the function of ETC complexes [6,7]. CTU was found to inhibit ETC complex III activity in tumour cells, which increased ROS production [8]. The resultant ROS activated the PKR-like endoplasmic reticulum (ER) kinase pathway of ER stress, which enables mitochondrial-ER cross-talk [8]. This, in turn, altered the expression of the BH3-only proteins NOXA and Mcl-1, which disrupted the OMM and facilitated the release of mitochondrial cytochrome c to promote apoptotic cell death [8]. However, cytochrome c is bound primarily in the IMM, and it has been shown that selective disruption of the OMM is not sufficient for the activation of apoptosis [9]. IMM disruption is also required to enable cytochrome c release [9]. Accordingly, the mechanism by which CTU disrupts the IMM to promote cytochrome c release and the activation of apoptosis is presently unclear.

Mitochondrial fusion maintains the quality and morphology of mitochondrial cristae, which are essential for ATP production [10]. IMM fusion is regulated by dynamin-related GTPase optic atrophy 1 (OPA1) [11]. The unprocessed and N-terminal transmembrane-anchored long form is termed L-OPA1, and the proteolysed short forms, which lack the transmembrane anchor, are termed S-OPA1 [12]. When the mitochondrial membrane potential is lost, L-OPA1 is cleaved to S-OPA1 isoforms by the stress-activated zinc metallopeptidase OMA1, leading to mitochondrial fragmentation [12]. In addition, it has been shown that mitochondrial fragmentation occurs with the loss of the mitochondrial membrane potential and with increased ROS formation [13]. Thus, the OMA1/OPA1 pathway is a critical regulator of IMM fusion.

The present study was undertaken to clarify the relationship between CTU-mediated mitochondrial disruption, ROS production and the activation of apoptosis. The principal finding to emerge was that CTU-mediated ROS production selectively dysregulated OMA1 and OPA1, which control IMM fusion. Thus, the loss of IMM fusion capacity is implicated in the loss of mitochondrial integrity in CTU-treated cells and the activation of apoptosis.

## 2. Results

### 2.1. Mitochondrial Disruption, ROS Production, and Decreased Viability in CTU-Treated Cells

**Figure 1:**
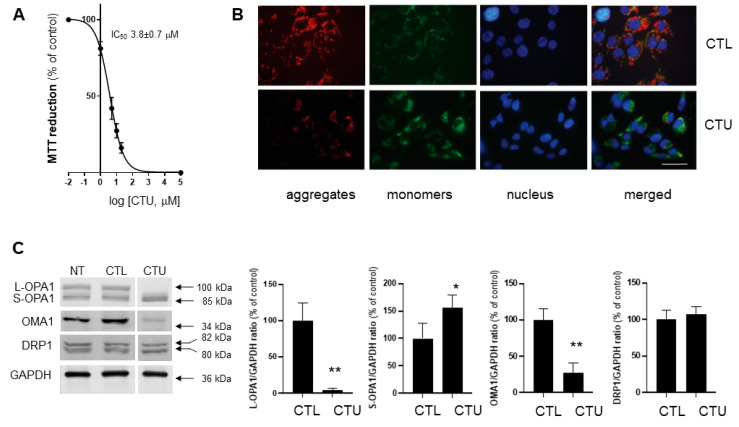
CTU treatment of MDA-MB-231 cells depolarises the mitochondrial membrane, disrupts the fusion mediator system of the inner mitochondrial membrane (IMM), and decreases cell proliferation. (**A**) Concentration-dependent inhibition of 3-(4,5-dimethylthiazol-2-yl)-2,5-diphenyltetrazolium bromide (MTT) reduction in CTU-treated MDA-MB-231 cells (24 h; log_10_[CTU] versus % of control activity). Control cells were treated with DMSO (0.1%) alone. (**B**) Confocal imaging of 5,5′,6,6′-tetrachloro-1,1′,3,3′-tetraethylbenzimidazolylcarbocyanine iodide (JC-1) in CTU-treated (10 μM; 4 h) and DMSO-treated (CTL) MDA-MB-231 cells. JC-1 dimers fluoresce red and monomers fluoresce green and indicate intact and disrupted mitochondria, respectively; Hoechst 33342 counter-staining was used to detect nuclear DNA in cells (blue). Merged images show overlap of JC-1 and Hoechst 33342-stained sections (scale bar: 20 μm). Confocal analysis was performed using a Zeiss LSM 800 instrument, as described in the Materials and Methods. (**C**) Impaired expression of the long form of optic atrophy 1 (L-OPA1) and the OMA1 zinc metallopeptidase (OMA1), increased expression of the short form of optic atrophy 1 (S-OPA1), and unchanged expression of dynamin-related protein 1 (DRP1) in CTU-treated cells (10 μM; 6 h). Immunoblots for proteins were obtained from the same membrane. Spaces between the blots shown in the figure indicate that signals were from non-adjacent lanes. All experiments were conducted on three separate occasions. The data are expressed as means ± SD. Different from CTL (DMSO alone): * *p* < 0.05 and ** *p* < 0.01 (unpaired Student’s *t*-test). NT: cells received no treatment.

In preliminary time-course experiments, alterations in early mechanistic events (mitochondrial membrane potential and lipid peroxidation), regulatory protein expression, and functional endpoints (cell proliferation and apoptosis) were maximal 4 h, 6 h and 24 h after CTU addition. Thus, it was not feasible to conduct all assays at a single time-point. Assays were conducted at optimal time-points after CTU addition based on preliminary experiments. As shown in Figure 1A, CTU decreased the viability of MDA-MB-231 cells in a concentration-dependent fashion. Thus, MTT reduction was decreased with an IC_50_ of 3.8 ± 0.7 μM. Consistent with previous findings [14], CTU (10 μM; 24 h) also increased apoptosis, as reflected by the caspase-3/7 activity, to 3.5 ± 0.6-fold of the control (*p* < 0.01; Figure S1). CTU also effectively depolarised the mitochondrial membrane, as indicated by the concentration-dependent decrease in the JC-1 red–green fluorescence ratio (IC_50_ 4.5 ± 1.4 μM; Figure S2). This finding is consistent with the decrease in JC-1 aggregates (red fluorescence) and the increase in JC-1 monomers (green fluorescence) observed using confocal microscopy (Figure 1B); Hoechst 33342 counter-staining was used to detect nuclear DNA in cells. Together, these findings indicate that mitochondrial membrane depolarisation by CTU (10 μM) also occurred with an increase in ROS-mediated production of lipid peroxides, as detected with the dye BODIPY (581/591) C11 (*p* < 0.01; Figure S3).

### 2.2. CTU Dysregulates the OMA1/OPA1 Pathway of IMM Fusion in Cells

The potential involvement of IMM fusion in CTU-mediated apoptosis was evaluated in MDA-MB-231 cells. The cellular expression of the IMM fusion regulatory mediators OPA1 and OMA1 was assessed using Western immunoblotting. As shown in Figure 1C, the expression of L-OPA1 was markedly decreased to 3.9 ± 3.0% of the control following treatment with CTU (10 μM; 6 h; *p* < 0.01). In contrast, the expression of the S-OPA1 short isoforms was increased to 155 ± 23% of the control (*p* < 0.05; Figure 1C). Thus, CTU decreased the L-OPA1/S-OPA1 expression ratio in cells. The metalloendopeptidase OMA1 has a central role in mediating the proteolytic cleavage of L-OPA1 to S-OPA1 [15]. As shown in Figure 1C, CTU treatment (10 μM; 6 h) markedly decreased OMA1 expression to 28 ± 13% of the control (*p* < 0.01). In comparison, CTU (10 μM; 6 h) did not alter the expression of the major mitochondrial fission regulator DRP1 (Figure 1C). Thus, treatment of MDA-MB-231 cells with CTU selectively disrupted the dynamic regulation of the IMM fusion mediators OPA1 and OMA1.

### 2.3. Role of CTU-Derived ROS in Dysregulation of OMA1-OPA1

**Figure 2:**
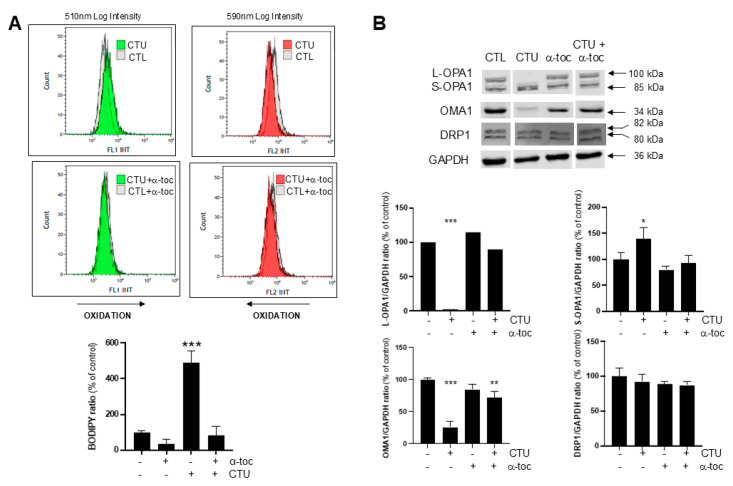
CTU-derived reactive oxygen species (ROS) disrupt the IMM fusion system. (**A**) The increase in ROS production in MDA-MB-231 cells mediated by CTU (10 μM; 4 h) that was detected using the (T-4)-difluoro [5-[[5-[(1E,3E)-4-phenyl-1,3-butadien-1-yl]-2H-pyrrol-2-ylidene-κN]methyl]-1H-pyrrole-2-undecanoato(2-)-κN^1^]-borate(1-), monohydrogen (BODIPY (581/591) C11) dye ratio was attenuated by the pretreatment of cells with α-tocopherol (α-toc; 100 μM; 4 h). Arrows show the shifts produced by CTU. (**B**) CTU-mediated decreases in L-OPA1 and OMA1 and increases in S-OPA1 were normalised by α-tocopherol pretreatment (100 μM; 4 h). Immunoblots for proteins were obtained from the same membrane. Spaces between the blots shown in the figure indicate that signals were from non-adjacent lanes. All experiments were conducted on three separate occasions. The data are expressed as means ± SD. Data from experiments with multiple treatments were analysed using one-way ANOVA in combination with Tukey’s post hoc test. Different from CTL: * *p* < 0.05, ** *p* < 0.01, and *** *p* < 0.001.

Previous studies have suggested that ROS impair OPA1 oligomerisation and OMA1 expression [15,16]. To assess the potential role of ROS in the dysregulation of OPA1 and OMA1 expression by CTU, co-treatment experiments were undertaken with the lipid phase peroxyl radical scavenger α-tocopherol. As anticipated, in initial studies with the dye BODIPY (581/591) C11, pretreatment of cells with α-tocopherol (100 μM; 4 h) attenuated ROS production in CTU-treated cells; α-tocopherol alone did not alter basal ROS (Figure 2A). In the present study, the effect of α-tocopherol on OPA1/OMA1 expression in CTU-treated MDA-MB-231 cells was assessed by Western immunoblotting. Following pretreatment with α-tocopherol (100 μM; 4 h) the dysregulation of L-OPA1, S-OPA1, and OMA1 expression in CTU-treated cells was prevented (Figure 2B); α-tocopherol did not alter DRP1 expression. These findings support the central role of mitochondrial ROS in the dysregulation of OPA1/OMA1 fusion mediators by CTU.

**Figure 3:**
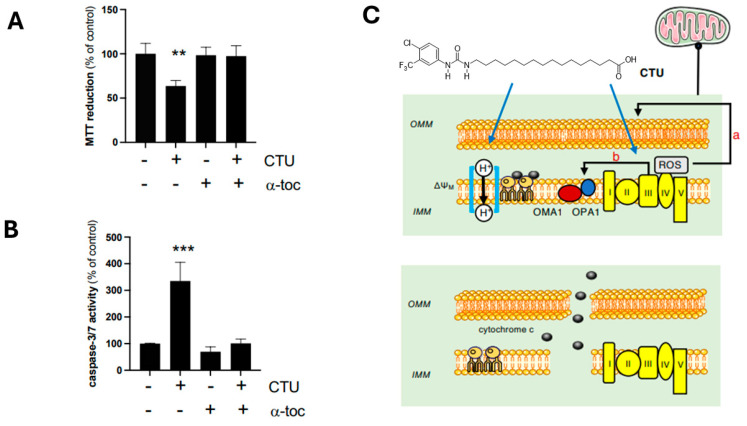
Functional significance of CTU-derived ROS and IMM disruption. The (**A**) decrease in MTT reduction and (**B**) increase in caspase-3/7 activity produced by CTU (10 μM; 24 h) were normalised by pretreatment of cells with α-tocopherol (α-toc; 100 μM; 4 h). (**C**) Mitochondrial targeting and CTU-mediated ROS production disrupts the outer mitochondrial membrane (OMM; path a), and the OMA1 zinc metallopeptidase/optic atrophy 1 (OMA1/OPA1) fusion regulatory system at the inner mitochondrial membrane (IMM; path b) enables cytochrome c release that activates apoptosis. The upper panel shows that the initial protonophoric uncoupling (proton transport across the IMM) and complex III inhibition (ETC complexes I–V) promotes ROS production. ΔΨ_M_ indicates mitochondrial membrane potential. The lower panel shows the disrupted IMM and OMM and the release of cytochrome c. All experiments were conducted on three separate occasions. The data are expressed as means ± SD. Data from experiments with multiple treatments were analysed using one-way ANOVA in combination with Tukey’s post hoc test. Different from CTL: ** *p* < 0.01 and *** *p* < 0.001.

In support of these findings, pretreatment of cells with α-tocopherol also restored the viability of CTU-treated cells. Thus, α-tocopherol (100 μM; 4 h) prevented the decrease in MTT reduction activity (Figure 3A), the increase in caspase-3/7 activity (Figure 3B), and the expression of cleaved caspase-3 immunoreactive protein in CTU-treated MDA-MB-231 cells (Figure S4).

## 3. Discussion

The principal new finding arising from the present study is that the anticancer aryl-ureido fatty acid CTU dysregulates the IMM fusion regulators OPA1 and OMA1. Previous studies have established that CTU is a lipophilic protonophore that uncouples mitochondrial oxidative phosphorylation and decreases ATP production [5]. CTU also inhibits ETC complex III, which increases cellular ROS production. ROS then activate ER stress pathways that disrupt the OMM, promote cytochrome c release, and activate apoptosis [8]. However, this mechanism does not explain the release of cytochrome c, which is located primarily within the IMM. Indeed, it has been demonstrated that disruption of the OMM alone is not sufficient for the activation of apoptosis [9]. The present finding that IMM disruption is associated with the dysregulation of the IMM proteins OPA1 and OMA1 provides new insight into the activation of apoptosis by CTU.

In the present study, Western analysis showed that CTU treatment decreased L-OPA1 expression and increased the expression of S-OPA1 isoforms in MDA-MB-231 cells. OPA1 is an important link between mitochondrial structure and oxidative phosphorylation. When the potential across the IMM (ΔΨ_m_) is intact, OPA1 promotes fusion [15,16]. Loss of ΔΨ activates the metalloprotease OMA1, which converts L-OPA1 to S-OPA1 and promotes mitochondrial fragmentation [15,16]. Thus, certain mitochondrial ETC inhibitors, such as valinomycin, oligomycin, and carbonyl cyanide *m*-chlorophenylhydrazine, stimulate the cleavage of L-OPA1 to S-OPA1 [15,16]. Early studies suggested that the fusion activity of S-OPA1 isoforms was low [11,17]. However, more recent findings have shown that both L-OPA1 and S-OPA1 are important for mitochondrial fusion and for the reticulated morphology of the mitochondrion [18,19]. Mechanistically, S-OPA1 has been proposed to facilitate the assembly of the IMM fusion machinery and membrane remodelling [20]. The marked perturbation of the L-OPA1–S-OPA1 ratio in favour of S-OPA1 that was elicited by the anticancer lipid CTU is associated with dysregulation of IMM fusion capacity and mitochondrial integrity.

Cleavage of OPA1 is an important mitochondrial stress response mechanism and has emerged as a critical early indicator of oxidative stress [19]. Thus, after the depletion of ΔΨ, stress stimuli trigger mitochondrial fragmentation and the loss of oxidative phosphorylation and also activate OMA1 [15,16]. Positively charged amino acid residues in the N-terminal region are crucial for OMA1 activation that occurs after the dissipation of ΔΨ, which then promotes L-OPA1 proteolysis and mitochondrial fragmentation [15]. However, after activation, the OMA1 protein also undergoes autocatalytic degradation in the N- and C-terminal regions [12,21]. This enables the potential restoration of the mitochondrial network following the removal of stress stimuli or after mitochondrial damage is repaired. If the production of ROS or other stress stimuli persists, mitochondrial fragmentation results in cell death [17,22]. In the present study, CTU treatment markedly increased ROS production, as detected with the lipid peroxidation sensor BODIPY (581/591) C11, which enhanced apoptotic cell death.

The increased processing of L-OPA1 to S-OPA1 by activated OMA1 has emerged as a general response to cellular stresses [18]. Interestingly, it has been suggested that increased S-OPA1 production might be a pro-survival adaptation in oxidant-stressed cells and following ischemia–reperfusion injury [21,23]. Indeed, cells that are unable to generate S-OPA1 have been found to be more susceptible to cytotoxic stress [23]. Mechanistically, cells that express L-OPA1 exclusively generate larger quantities of superoxide and are more sensitive to mitochondrial permeability transition [23]. Importantly, silencing of OMA1 exacerbated oxidant-induced cell death [23]. The present findings suggest that the increase in S-OPA1 expression following CTU-mediated ROS production may be a pro-survival response [17].

The role of ROS in OPA1/OMA1 dysregulation was evaluated. It was found that the peroxyl radical scavenger α-tocopherol attenuated lipid peroxidation in CTU-treated cells and prevented the altered expression of OMA1. OMA1 is a redox-dependent protein and exists in a partially oxidised state in mitochondria [24,25]. Its activity and stability are modulated in a redox-sensitive manner by two cysteine residues, Cys^272^ and Cys^332^, which are exposed to the inter-membrane space [24]. OMA1 also performs protein quality control functions, including the stabilisation of ETC complexes, which influences oxidative phosphorylation [25,26]. However, the proximity of OMA1 to ROS generated by ETC complexes creates an environment with a high probability of protein malfunction [24]. Indeed, ETC complex III contains two redox-active sites that release ROS on either side of the IMM [27]. Moreover, it has been previously shown that selective inhibitors of both sites in complex III modulate ROS production in CTU-treated cells [8]. The location of OMA1 in relation to complex III could also be significant for the maintenance of mitochondrial integrity because fusion events occur at mitochondria–ER contact sites [28,29]. Thus, the protection of the OPA1/OMA1 system from CTU-mediated ROS-dependent dysregulation by α-tocopherol treatment also maintained MDA-MB-231 cell viability, as shown by the restoration of cell proliferation (MTT reduction activity) and the attenuation of apoptosis (caspase-3/7 activity).

## 4. Materials and Methods

### 4.1. Cell Culture Reagents and Chemicals

Human MDA-MB-231 breast cancer cells were purchased from the American Type Culture Collection (Manassas, VA, USA). Phosphate-buffered saline (PBS), Dulbecco’s modified Eagle’s medium (DMEM; low glucose) and 3-(4,5-dimethylthiazol-2-yl)-2,5-diphenyltetrazolium bromide (MTT) were purchased from Sigma Aldrich (Castle Hill, NSW, Australia). Foetal bovine serum (FBS), penicillin–streptomycin, absolute methanol, trypsin–EDTA, and (T-4)-difluoro [5-[[5-[(1E,3E)-4-phenyl-1,3-butadien-1-yl]-2H-pyrrol-2-ylidene-κN]methyl]-1H-pyrrole-2-undecanoato(2-)-κN^1^]-borate(1-), monohydrogen (BODIPY (581/591) C11) dye were obtained from Thermo Fisher Scientific (Mulgrave, VIC, Australia). The reagents for electrophoresis were purchased from Amresco (Solon, OH, USA) or Bio-Rad (Richmond, CA, USA). 5,5′,6,6′-Tetrachloro-1,1′,3,3′-tetraethylbenzimidazolylcarbocyanine iodide (JC-1) was acquired from Sapphire Bioscience (Redfern, NSW, Australia).

General analytical-grade laboratory chemicals and high-performance liquid chromatography-grade solvents were obtained from LabScan (Lomb Scientific, Taren Point, NSW, Australia) or Ajax Chemicals (Sydney, NSW, Australia). The ureido fatty acid analogue CTU (16({[4-chloro-3-(trifluoromethyl)phenyl]-carbamoyl}amino)hexadecanoic acid) was prepared as reported previously in [14].

The anti-OPA1 (catalogue number 612607) and anti-OMA1 (17116-1-AP) antibodies were obtained from Beckton Dickinson Biosciences (North Ryde, NSW, Australia) and ProteinTech (Rosemont, IL, USA), respectively. Anti-dynamin-related protein 1 (DRP1; 4E11B11) and anti-glyceraldehyde 3-phosphate dehydrogenase (GAPDH; 2118S), were purchased from Cell Signalling Technology (Arundel, QLD, Australia), while anti-β-actin (C47778) was purchased from Santa Cruz Biotechnology (Dallas, TX, USA). The anti-mouse IgG (DyLight^TM^ 680 Conjugate) and anti-rabbit IgG (DyLight^TM^ 800 4X PEG Conjugate) secondary antibodies were purchased from Cell Signaling Technology (Danvers, MS, USA).

### 4.2. MTT Reduction Assay of Cell Proliferation

MDA-MB-231 cells were maintained in DMEM low-glucose medium supplemented with 10% FBS and 1% penicillin–streptomycin. MTT reduction assays of cell proliferation were conducted as reported previously in [30]. Briefly, cells (7 × 10^3^ cells/well) were seeded in triplicate in 96-well microplates and incubated at 37 °C for 24 h. The serum was removed, and 24 h later, the cells were treated with CTU (1–20 µM; 24 h), and the rate of MTT reduction was measured with a Victor 3V 1420 multi-label counter (Perkin Elmer, Akron, OH, USA). Control cells were treated with the vehicle alone (DMSO; 0.1%). The IC_50_ values were determined by the non-linear regression of plots of log_10_ [CTU] versus the percentage of control activity using GraphPad Prism 8 (GraphPad Software Inc., San Diego, CA, USA).

### 4.3. Caspase-3/7 Assay of Cell Apoptosis

MDA-MB-231 cells were seeded in duplicate (7 × 10^3^ cells/well) in black-walled 96-well plates and incubated at 37 °C for 24 h. The medium was removed, and the cells were incubated for a further 24 h in a serum-free medium before treatment with CTU (10 μM) for 24 h. The luminescence was measured using the Caspase-Glo 3/7 assay of cell apoptosis, conducted according to the manufacturer’s instructions (Promega, Madison, WI, USA) with a SpectraMax iD5 microplate reader (Molecular Devices, San Jose, CA, USA).

### 4.4. JC-1 Fluorescence Assay of the Mitochondrial Membrane Potential

MDA-MB-231 cells were seeded in triplicate in 96-well microplates (7 × 10^3^ cells/well). The serum was removed, and 24 h later, the cells were treated with CTU (10 µM) for 4 h and then incubated with JC-1 (1.5 µM) in a serum-free medium for 20 min. Following serum removal, the cells were washed with PBS, and the fluorescence of the JC-1 aggregates and monomers was measured at excitation/emission wavelengths of 535/595 nm and 485/535 nm, respectively, with a Fluoroskan Ascent FL microplate reader, according to the manufacturer’s instructions (Labsystems, Upplands Väsby, Stockholm County, Sweden).

### 4.5. BODIPY (581/591) C11 Lipid Peroxidation Assay

MDA-MB-231 cells (7.5 × 10^4^/well) were seeded in duplicate in 6-well plates, and 24 h after serum removal, were treated with CTU (10 μM; 4 h). The cells were then incubated with BODIPY (581/591) C11 dye to detect lipid peroxidation (1 µM; 30 min). The cells were trypsinised, washed, and resuspended in PBS (500 µL) for analysis with a Gallios flow cytometer using the Kaluza version 2.1 software, according to the manufacturer’s instructions (Beckman Coulter, Lane Cove West, NSW, Australia).

### 4.6. Preparation of Cell Lysates for Electrophoresis and Immunoblotting

MDA-MB-231 cells were seeded (8 × 10^5^/well) in a 100 mm culture dish (Corning, Noble Park, VIC, Australia) and were cultured at 37 °C for 24 h. The cells were then incubated in a serum-free medium for a further 16 h and were then treated with CTU (10 μM) or DMSO (0.1%; control) for 24 h. The cells were harvested (at ~80–90% confluence) using trypsin–EDTA before lysis in 10 mM Tris buffer (pH 6.8) containing 150 mM NaCl, 0.5% sodium dodecylsulfate, 1% Triton X-100, 1 mM EDTA, and a protease/phosphatase inhibitor cocktail (Cell Signaling Technology). The protein lysates were subjected to electrophoresis on 7.5–12% SDS–polyacrylamide gels, as described previously in [31]. The separated proteins were transferred electrophoretically to 0.2 μm nitrocellulose membranes and subjected to Western immunoblotting, as described in [32]. The membranes were incubated overnight with primary antibodies in 1% bovine serum albumin, 10 mM Tris, 100 mM NaCl, and 0.1% Tween 20 (1:1000). The following day, the membranes were washed in 10 mM Tris, 100 mM NaCl, and 0.1% Tween 20 and then incubated with anti-rabbit or anti-mouse IgG secondary antibodies in 1% bovine serum albumin, 10 mM Tris, 100 mM NaCl, and 0.1% Tween 20 (1:10,000) at room temperature for 1 h. The membranes were then scanned using an Odyssey Infrared System with the Image Studio Light version 5.2.5 software (Li-Cor Biosciences, Lincoln, NE, USA).

### 4.7. Confocal Microscopy and Cell Staining for Mitochondrial Integrity and DNA

MDA-MB-231 cells (1.5 × 10^5^) were seeded on 35 mm Petri dishes. The cells were washed with PBS 24 h after serum removal and then treated with CTU (10 µM; 4 h). The cells were then incubated with JC-1 (1.5 µM) and Hoechst 33342 (8 µM) in a serum-free medium for 20 min. The fluorescence of dimeric and monomeric JC-1 was detected at excitation/emission wavelengths of 535/595 nm and 485/535 nm, respectively, and Hoechst 33342 using excitation/emission wavelengths of 361/497 nm, with a ZEISS LSM 800 confocal microscope in Airyscan mode, and analysed using the ZEN lite software (ZEISS, Oberkochen, Germany, https://www.zeiss.com/microscopy/en/products/software/zeiss-zen-lite.html, accessed on 27 September 2024).

### 4.8. Statistics

All experiments were conducted on at least three separate occasions. The data are expressed throughout as means ± standard deviations (SDs). Statistical analysis was performed using GraphPad Prism 8. The data from experiments with multiple treatments were analysed using a one-way analysis of variance (ANOVA) in combination with Tukey’s post hoc test. Statistical significance was defined as *p* < 0.05 for the overall analyses, with the actual differences between treatments indicated in the figure legends.

## 5. Conclusions

The principal finding arising from the present study is that the production of ROS by CTU disrupts the OMA1/OPA1 fusion system, which controls the integrity of the IMM (Figure 3C). Agents that selectively impair pathways that control mitochondrial dynamics in cancer cells have not been widely considered in drug development strategies. To our knowledge, CTU is the first agent that targets the IMM fusion machinery and also disrupts the mitochondrial OMM to activate apoptosis. Further studies will now be required to evaluate whether agents such as CTU have value in anticancer chemotherapy in patients.

## Data Availability

Data is contained within the article (and Supplementary Materials).

## Supplementary Materials

The following supporting information can be downloaded at https://www.mdpi.com/article/10.3390/ijms251910577/s1.
