# Supplementary material for: The Novel Anticancer Aryl-Ureido Fatty Acid CTU Increases Reactive Oxygen Species Production That Impairs Mitochondrial Fusion Mechanisms and Promotes MDA-MB-231 Cell Death
Source: Int J Mol Sci. 2024 Oct 1;25(19):10577. doi: 10.3390/ijms251910577 (PMC11476390; doi:10.3390/ijms251910577)
Supplement: Supplementary file 1 [file ijms-25-10577-s001.zip › ijms-3204475-supplementary.pdf]

Supplementary information

Stanton Tam 1 , Balasubrahmanyam Umashankar 1,† , Md Khalilur Rahman 1,‡, Hassan Choucair 1,§ ,

Tristan Rawling 2 and Michael Murray 1,*

1 Pharmacogenomics and Drug Development Group, Sydney Pharmacy School, Faculty of Medicine and Health, University of Sydney, Sydney, NSW 2006, Australia; stanton.tam@anu.edu.au (S.T.); b.umashankar@unsw.edu.au (B.U.); khalilur@mucpharm.com (M.K.R.); hassan.choucair@nd.edu.au (H.C.)

2 School of Mathematical and Physical Sciences, Faculty of Science, University of Technology Sydney, Ultimo, NSW 2007, Australia; tristan.rawling@uts.edu.au

*** Correspondence: michael.murray@sydney.edu.au**

† Current address: Molecular and Integrative Cystic Fibrosis Research Group, School of Biomedical Sciences, Faculty of Medicine and Health, University of New South Wales, Sydney, NSW 2052, Australia.

‡ Current address: Mucpharm Pty Ltd., Kogarah, Sydney, NSW 2217, Australia.

§ Current address: School of Health Sciences, Faculty of Medicine, Nursing and Midwifery and Health Sciences, University of Notre Dame Australia, Chippendale, NSW 2007, Australia.

Includes: Figures S1-S4

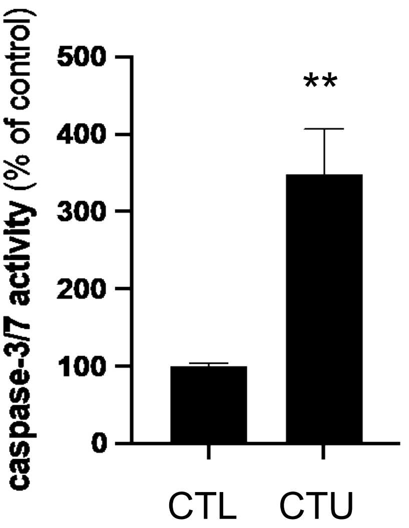

Figure S1: Increased caspase-3/7 activity produced in MDA-MB-231 cells by CTU. MDA-MB-231 cells were seeded in duplicate (7 x 10^3 cells/well) in black-walled 96-well plates and incubated at 37°C for 24 h. The medium was removed and cells were incubated for a further 24 h in serum-free medium before treatment with CTU (10 μM) for 24 h. Luminescence was measured using Caspase-Glo 3/7 assay according to the manufacturer's instructions (Promega, Madison, WI) in a SpectraMax iD5 microplate reader (Molecular Devices, San Jose, CA). Experiments were conducted on three separate occasions. The data are expressed as means ± standard deviations. Different from control (CTL): **P<0.01 (unpaired Student's t-test).

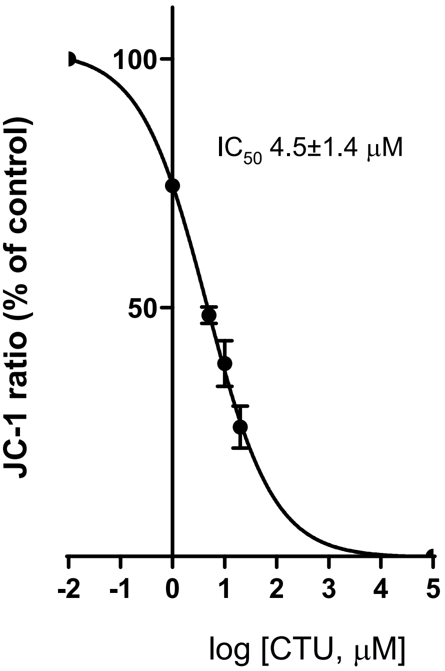

Figure S2: MDA-MB-231 cells were seeded in triplicate on 96-well microplates (7 x 10^3 cells/well). Twenty-four h after serum removal, cells were treated with different concentrations of CTU for 4 h, then incubated with JC-1 (1.5 μM) in serum-free medium for 20 min. Following serum removal, cells were washed with PBS and the fluorescence of JC-1 aggregates and monomers was measured at

excitation/emission wavelengths of 535/595 nm and 485/535 nm, respectively, in a Fluoroskan Ascent FL micro-plate reader (Labsystems, Upplands Väsby, Stockholm County, Sweden). The IC_{50} was determined by non-linear regression of the plot of log_{10} [CTU] versus % of control JC-1 ratio (GraphPad Prism 8).

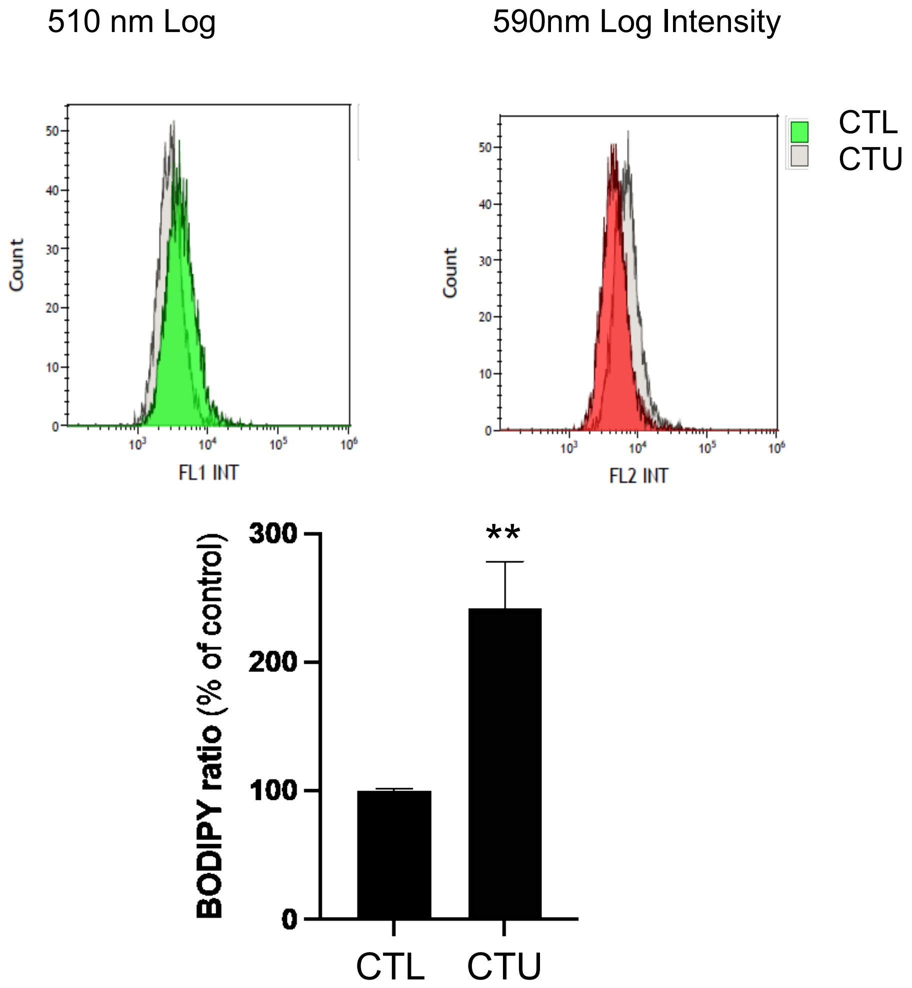

Figure S3: ROS production in MDA-MB-231 cells after treatment with CTU (10 µM, 24 h) was detected using C11-BODIPY (581/591). MDA-MB-231 cells (7.5 x 10^4/well) were seeded in duplicate on 6-well plates and, 24 h after serum removal, were treated with CTU (10 µM, 4 h). Cells were then incubated with BODIPY (581/591) dye (1 µM, 30 min). Cells were trypsinized, washed and resuspended in PBS (500 µL) for analysis in a Gallios flow cytometer using Kaluza software (Beckman Coulter, Lane Cove West, NSW, Australia). Experiments were conducted on three separate occasions. The data are expressed as means ± SD. Different from CTL: **P<0.01 (unpaired Student's t-test).

.

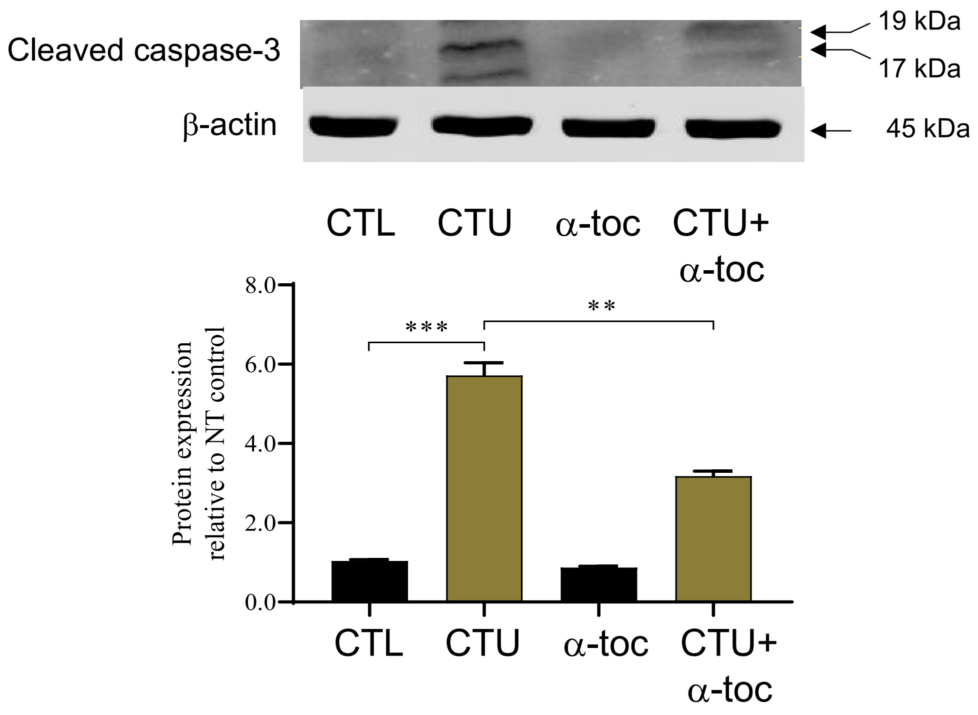

Figure S4: CTU-mediated increases in immunoreactive cleaved caspase-3 were normalized by pretreatment of MDA-MB-231 cells with α-tocopherol (α-toc, 100 μM, 4 h). Experiments were conducted on three separate occasions. The data are expressed as means ± SD. Data were analysed using one-way ANOVA in combination with Tukey's post hoc test. Different from CTU alone: **P<0.01, ***P<0.001.
